# Evaluating the experiences of Vietnamese university students enrolled in a longitudinal research cohort: a journey mapping approach

**DOI:** 10.3389/fmed.2026.1745499

**Published:** 2026-06-08

**Authors:** Hang Thi Thu Nguyen, Hung Bao Vu, Kien Duy Vu, Chi Phuong Le, Nguyet Minh Nguyen, Tam Thi Hoai Dong, Trung Dinh The, Thuy Thi Van Nguyen, Katrina Lawson, Mary Chambers, Dung Do Van, Tuan Diep Tran, Evelyne Kestelyn, Jennifer Ilo Van Nuil, Bridget Wills

**Affiliations:** 1 Oxford University Clinical Research Unit, Hospital for Tropical Diseases, Ho Chi Minh City, Vietnam; 2 Centre for Tropical Medicine and Global Health, University of Oxford, Oxford, United Kingdom; 3 University of Medicine and Pharmacy at Ho Chi Minh City, Ho Chi Minh City, Vietnam

**Keywords:** evaluation, experience, health professions students, health research, journey mapping, Vietnam

## Abstract

**Introduction:**

Engaging young people in health research offers mutual benefits for both participants and research teams. While a variety of approaches have been used globally to involve youth, few studies have evaluated their effectiveness and impact.

**Methods:**

From 2020 to 2023, we conducted the SEED project to explore attitudes toward clinical research involving human participants among 539 Vietnamese health professions students. The project concluded with an evaluation of the students’ experiences as project participants, using a mixed-methods journey mapping approach. For this we conducted in-depth exit interviews with 18 purposively selected students and analyzed 231 study completion questionnaires, as well as 815 feedback forms collected during the life of the project.

**Results:**

Findings are presented in three areas: (1) Students’ motivations to join and views about their experiences as participants; (2) Their evaluation of organizational factors; and (3) Their overall views about the project and scientific research generally. As well as having an interest in research (195/231, 84%), for many students the potential for personal benefit, such as gaining new knowledge (161/231, 70%), enhancing soft skills (161/231, 70%), and exposure to networking opportunities (126/231, 55%), were major motivating factors. However, factors such as receiving a project attendance certificate, the financial compensation offered, the reputation of the research institution, and encouragement by friends, were not considered important by many students (42–46%). Among students who attended at least one event, 217/220 (99%) reported high satisfaction with the project implementation, engagement approaches and activity topics, and generally preferred offline to online events. Suggested improvements included incorporating more interactive games, using smaller discussion groups, and strengthening communication via social media to support retention. 145/231 (63%) of the students mentioned at least one barrier to participation, with a busy schedule being the most common(127/145, 87%). For some of the interviewed participants, altruistic motivation– the sense of contributing to science and community–influenced their continuing participation.

**Conclusion:**

This comprehensive participant-centered evaluation of the experiences of Vietnamese healthcare students enrolled in a longitudinal cohort study emphasizes the value of engaging with students to co-develop scientific activities, thereby providing important benefits for the students, the academic institutions, and the broader research community.

## Introduction

The United Nations Population Fund estimates that young people aged between 10 and 24 years now account for a quarter of the world’s population (1). In recent years there has been increasing recognition of the potential power of this group to catalyze radical conversations and advocate for novel approaches to globally important problems (e.g., health-related issues, climate change, gender inequality, the right to education) (2–5). Research activities focused on this demographic that encourage active involvement—for example, a co-creation approach to study design, data synthesis, or result dissemination—can offer mutual benefits (3, 6). For the young people, participation can enhance knowledge, soft skills, and network development, which may in turn influence their subsequent career choices or motivate their involvement in efforts to address important health and social issues (7). At the same time, the research team is more likely to obtain representative data and be effective in promoting the research findings to the youth population, potentially increasing overall impact in relevant communities (7, 8).

A range of different approaches have been used to explore young people’s thoughts and attitudes toward research. Science workshops, science clubs, and science media publications are often utilized to connect researchers in a general way with large numbers of young people (9). Conversely, more formal research methods typically involve small groups in the discussion, training, execution, and evaluation processes, but such efforts rely on rigorous experimental design and can be time-consuming and expensive (10). Although approximately 90% of young people live in low and middle-income countries (LMICs) where disease burden is typically high, to date most formal studies related to youth participation in health research have been conducted in high-income countries (HICs) (1, 3, 10–13). Many factors contribute to this disparity, including constraints in terms of capacity, funding, or coordination by local research institutions, often compounded by lack of support from relevant government agencies (9, 10, 14, 15). However, LMIC-based efforts to involve young people in research related to infectious diseases and climate change are now gaining traction (10, 15). The majority of such projects do not, as yet, disseminate the results of their activities widely, nor examine the impact of young people’s involvement on their research outcomes (9).

Whatever the context, it is clear that in order to develop effective ways to communicate and interact with young people, build trust, and successfully leverage their contributions to improve research quality, a clear understanding of the motivations, needs and challenges of the relevant youth population is needed (10, 15, 16). Although the importance of effective engagement with all stakeholders prior to commencing any research endeavor is well established, the need for subsequent multi-disciplinary critical reflection and evaluation of participant experiences, in order to better inform future efforts, is a relatively recent concept (9, 14, 15). One approach that has proved useful in evaluating participant experience in areas such as medical education (17–19), and health services research (20–22), is journey mapping.

Journey mapping combines visualization with qualitative and quantitative approaches to understand an individual’s experiences over the course of a program or lengthy organized activity. The process aims to provide both a user-centric summary of that individual’s experiences as well as a broader overview of the study group’s collective experiences (23). Compared with traditional in-depth interviews that rely on semi-structured guidance and questions, integrating a visualization tool into the process allows participants to lead the discussion, making it particularly useful for exploratory research. The journey mapping technique is intended to help participants organize their thoughts and memories on paper, stimulating their recollection of key moments and providing an understanding of how earlier events may have influenced later perceptions and decisions. When used in conjunction with in-depth interviews, participants are able to refer back to elements of the map during the interview, enabling deeper exploration of their experiences and helping to clarify information gaps and capture key evaluation domains.

Vietnam, an LMIC in Southeast Asia with a population of approximately 100 million people in 2023 (24), faces major health and economic burdens related to both communicable and non-communicable diseases, especially in large cities such as Ho Chi Minh City (HCMC) and Ha Noi (25). The University of Medicine and Pharmacy at HCMC (UMP) is one of the foremost medical schools in the country; the university trains more than 600 medical and public health graduates each year (26), and is active in promoting the importance of scientific research within the medical community. The Oxford University Clinical Research Unit (OUCRU) has been conducting clinical and laboratory research related to tropical infectious diseases in HCMC since 1991, with public engagement and social science research subsequently incorporated into the program. In 2020, the OUCRU Dengue, Social Science, and Public Engagement groups, working in collaboration with the Faculties of Medicine and Public Health at UMP, began a program of work (the SEED project) that was designed to explore the perceptions and attitudes of students undertaking degrees in the health professions (medicine, public health, nutrition) regarding research involving human participants. We developed a prospective longitudinal cohort of students enrolled at the university, with written informed consent provided by all participants, and used a mixed-methods approach to investigate their views on a broad range of topics related to the conduct of clinical research, focusing in particular on dengue research.

The cohort was active for over 3 years, and during the last few months we elected to conduct an evaluation study designed to explore the students’ experience as participants in the SEED project. We were particularly interested to examine the factors that motivated students to join the project, as well as to explore their views on the different types of engagement and data collection activities that they attended. However, conducting an evaluation at the end of a project that had lasted for several years has limitations, regarding for example, accuracy of recall and/or delayed emotional reflection about particular events. Therefore, we chose to integrate multiple methods in our evaluation, including: (a) journey mapping and exit interviews with selected students, (b) a study completion questionnaire designed to explore students’ views more generally across the cohort, and (c) examination of post-event feedback forms that had been completed immediately after many of the project activities, with the overall aim of capturing a comprehensive understanding of the students’ experiences throughout their participation in the project.

## Materials and methods

### Project summary

The SEED project used an approach comprising initial deliberative engagement, a technique designed to educate and inform participants in a balanced non-directed way about complex topics, combined with both qualitative and quantitative data-collection methods, to explore the cohort participants’ thoughts and ideas on a series of research themes. For the engagement activities we used multiple formats including science cafés, science debates, role-plays, and poster/video competitions; for most of these events 30–50 students were selected from among the cohort participants who applied to attend, but there were also occasional open-access events and some specialized invitation-only events. The various engagement activities were organized in parallel with formal social science research methods including surveys, focus group discussions (FGDs), and in-depth interviews (IDIs). The detailed design and methodology of the SEED project are described elsewhere (27).

### Design of the evaluation study

For the evaluation study, we also employed a mixed-methods design, using a number of tools designed to capture students’ experiences and perceptions on the full range of project topics and activities at the end of their involvement after 3 years of the cohort. The key component of the evaluation study was a series of in-depth exit interviews incorporating journey mapping, that we conducted with small numbers of students randomly selected from each of several groups, where group membership was defined by the level of participation in project activities. To confirm and further explore the cohort experience at a broader scale, we subsequently invited all project participants to fill out a study completion questionnaire. Finally, to provide more timely and detailed evaluation of specific engagement formats, we also analyzed all available post-event feedback forms. Students attending any activity throughout the life of the project had been encouraged to complete these forms immediately afterwards, thus potentially mitigating any recall bias associated with retrospective reporting in the exit interviews and completion questionnaires.

### Sampling and data collection tools

#### Journey mapping/exit interviews

For this first component of the evaluation, we used random sampling to select participants for the journey mapping and exit interviews, focusing on students who had joined at least one project activity. To ensure we gathered a range of opinions from students with different levels of involvement in the project, we first stratified students into three similar-sized groups based on the number of events they had attended: Group 1 (G1) 1–2 events, Group 2 (G2) 3–5 events, and Group 3 (G3) ≥ 6 (maximum 16) events. Students who had never participated in any of the activities were designated Group 0 (G0). We aimed to recruit six students from each of Groups 1–3. These students were selected by simple random sampling within the Groups and invited to an interview by phone, text message, or email. If a student declined to participate, the next randomly selected student in the same Group was invited to an interview. The interviews were conducted either online or offline, depending on the students’ preference. Each interview was facilitated by one or two of three research team members with training in social science and public engagement methodology. As with other SEED activities, each student was reimbursed the equivalent of 10 USD for their time and effort to join the interview.

We developed a two-part process for the interviews. During the first part, the students were supported to develop their own individual journey map, i.e., to visualize their participation and experiences during the life of the project in chronological order. Each student created their personal map on a blank piece of paper, with a horizontal axis representing the timeline of their participation, and a vertical axis indicating their positive or negative experience of each event. From a set of sticky notes naming the different types of project activities, the student chose activities that they had attended and placed these notes on the map based on the timing of the event and a relative rating for their positive or negative experience. They could also use other blank notes to add more detail about each activity, describing the platform, format, topic, their memories or experience, etc. More detail of the exit interview guidance and an example of a journey map are presented in Supplementary Appendix 1.

The second part comprised a semi-structured interview. Based on the individual’s personal journey map, the interviewers asked follow-up questions to explore the student’s positive or negative experiences of the events they had attended, what made the events memorable, what they had learned, the impact of the event format on their participation etc. The students were also asked a series of specific questions about their motivations to join the project, their expectations and experiences, any barriers or difficulties encountered, their evaluation of organizational factors, and finally their views on benefits from participation and the project’s impact on their perceptions about science.

The interview guidance was piloted with two randomly selected students from G3 to test questions across the different activity formats that they had attended. Based on the pilot assessments, we refined how the students were asked to rate their experiences when creating their journey map, and made minor revisions to the wording of the specific interview questions.

#### Completion questionnaires

After all the exit interviews had been conducted and prior to the end of the main project, we developed a study completion questionnaire, aimed at exploring the students’ experiences at the cohort level. The structure and content of the questionnaire was based on the main topics discussed in the exit interviews by the 18 selected students, and so no pilot testing was carried out. The questionnaire included 8 multiple choice questions regarding motivations to join the project, overall assessments of the various activities using a Likert-5 scale, questions about barriers to participation, and one open-ended question for other comments. Detailed information of the completion questionnaires is presented in Supplementary Appendix 2. For students in G0, the questionnaire did not include the activity assessment section. All cohort participants were encouraged to complete this questionnaire; it was distributed both offline at the final event that everyone was invited to attend, as well as online on the project social media platforms for a 2-month period. Understanding that students in G0 might neither participate in the final event nor follow the relevant social media platforms, we sent the questionnaire directly to these students twice via their personal and institutional email addresses, specifically aiming to increase the response rate from this group.

#### Post-event feedback forms

These feedback forms had been collected before the evaluation study was conceived or set up. Immediately following most of the cohort activities undertaken during the 3-year life of the project (excluding open-access and invitation-only events), participants were offered the opportunity to complete a short feedback form. As well as structured questions designed to explore the student’s assessment of the specific activity, the forms included free text sections for general comments and suggestions for improvement in subsequent events. Detailed information of the post-event feedback forms is presented in Supplementary Appendix 3.

### Data analysis

We used descriptive statistics (number, percentage) to summarize categorical data collected in the surveys. We managed the qualitative data in NVivo12 software and used a form of thematic analysis, with an inductive approach (28–30). This approach allowed us to capture detailed descriptions of the students’ experiences, as well as providing flexibility to identify unexpected aspects mentioned by participants during the exit interviews or recorded on the post-event feedback forms.

All the forms/questionnaires and interviews were completed in Vietnamese. The interviews were also transcribed, coded, and analyzed in Vietnamese by one particular individual from among the three interviewers (the analyst). Based on the interview topics and preliminary coding for the two pilot interviews, all three interviewers and the senior investigators discussed the approach to develop and finalize the coding frame (main categories, sub-categories, initial codebooks) and coding procedure. Using the coding frame, all 18 interviews (including the two pilot interviews) went through two rounds of coding. The first round included data familiarization and open coding, to allow for adjustments to the codebook if necessary. The interview transcripts were also sorted based on demographic characteristics [e.g., gender, academic year, faculty, attendance Group (1–3)] to identify patterns or differences between sub-groups. To enhance consistency of the analysis conducted by a single individual, the second round of coding was conducted after 2–3 months, when the first round of coding had been completed for all interviews. The second round of coding included re-reading, re-arranging the first-round coded data into different sub-categories if needed, dividing the codes into smaller units, and re-sorting the codebooks into sub-categories. Quotes cited in the paper were translated into English by the analyst and reviewed by a senior researcher in the team. The analyst and the reviewer then discussed addressing any disagreement in the translation to ensure the quotes and interpretation correctly reflected the students’ meaning in the context of the project.

The final codebook developed from the exit interviews was also used to code the qualitative data from the post-event feedback forms. The final categories and sub-categories were used to develop the results framework presented in this paper.

## Results

Between 2020 and 2023, 539 students were recruited to the SEED project, including 365 (68%) and 174 (32%) students enrolled at the faculties of General Medicine and Public Health respectively. Among the 539 students, 530 participants variously attended 48 engagement activities, 24 FGDs, 19 IDIs, and/or completed at least one study questionnaire prior to the start of the project evaluation process. The remaining 9 students did not interact with project staff at all. During the initial phase of the project all activities were conducted in-person, but between July 2021 and January 2022, most activities were transferred to on-line formats to comply with societal restrictions imposed in response to the COVID-19 pandemic.

**FIGURE 1:**
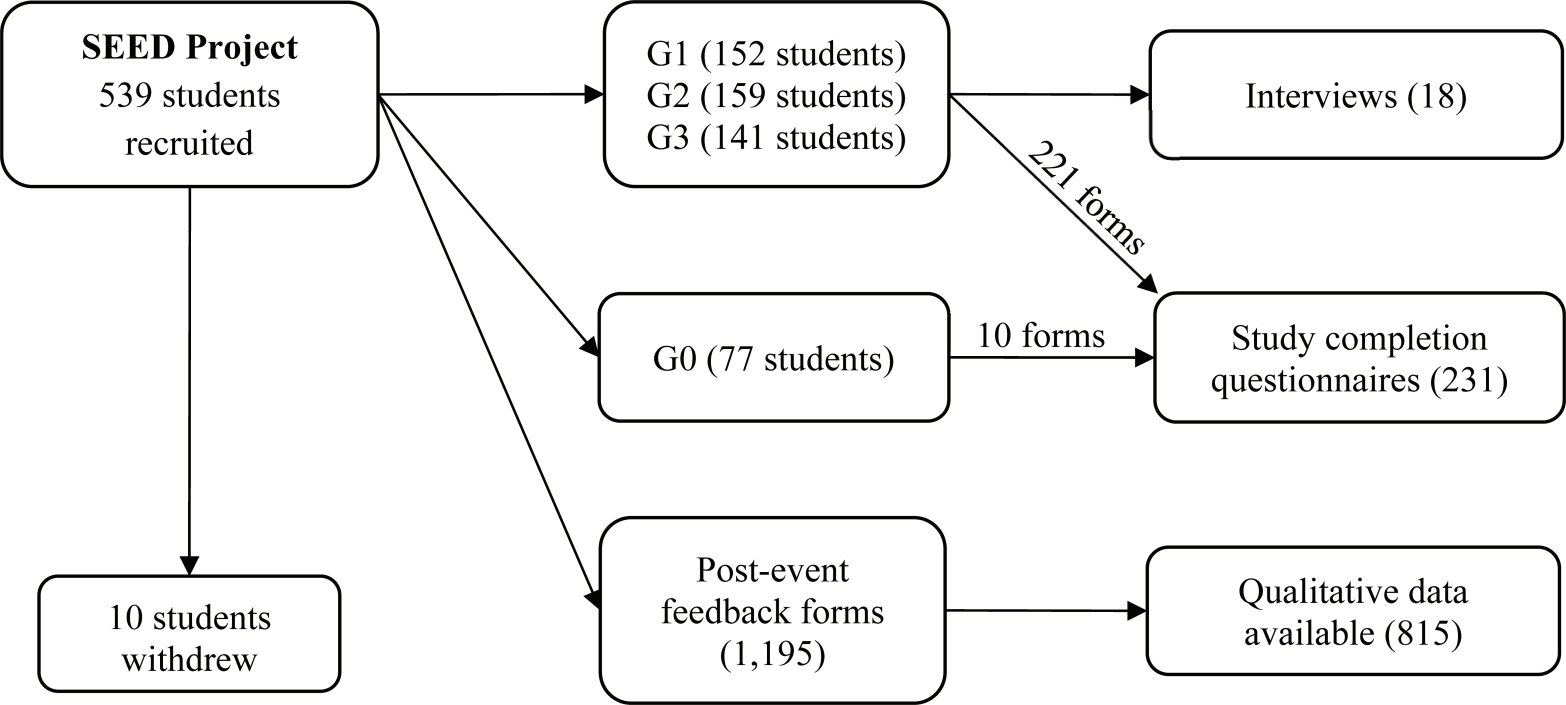
Flowchart summarizing the data sources available for this analysis.

Ten students (2%) withdrew from the project, and 77 (14%) students never joined any event (Group 0). The remaining 452 students were reasonably evenly distributed between attendance Groups 1–3 (Figure 1). All students in Groups 2 and 3 accepted the exit interview invitation, but 14 invitations were needed to achieve 6 exit interviews with Group 1 participants (43% acceptance rate). The 18 students who attended these interviews (12 via Zoom, 6 in person) were reasonably representative of the SEED cohort overall, in terms of gender, faculty and academic year (Supplementary Appendix 4).

We received 247 study completion questionnaires during the 2-month period. Of these, 16 responses had to be excluded for administrative reasons (duplication, student ID code missing). Therefore, for the final analysis, 231 study completion questionnaires, were available including 221 from students in Groups 1–3 and 10 from Group 0 students. In addition, following the 40 engagement activities after which students were offered the opportunity to complete a post-event feedback form a total of 1,195 forms were submitted; among these 815 contained some free text that was included in the qualitative data analysis.

**FIGURE 2:**
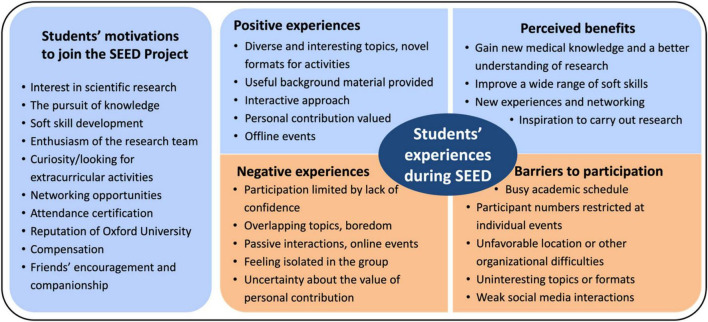
Commonly reported factors influencing students’ journeys as participants in the SEED project.

Based on analysis of these various data sources, we present our findings in three main areas: (1) Students’ motivations to join the SEED project and views about their experiences as participants; (2) Their evaluation of the practical organization of the cohort and the events they attended; and (3) Their overall views on the project, and on scientific research in general. A summary diagram indicating factors that could influence a student’s decision to participate and/or their overall experience of involvement in the SEED Project is summarized in Figure 2.

### Motivations, experiences, and perceived benefits

**FIGURE 3:**
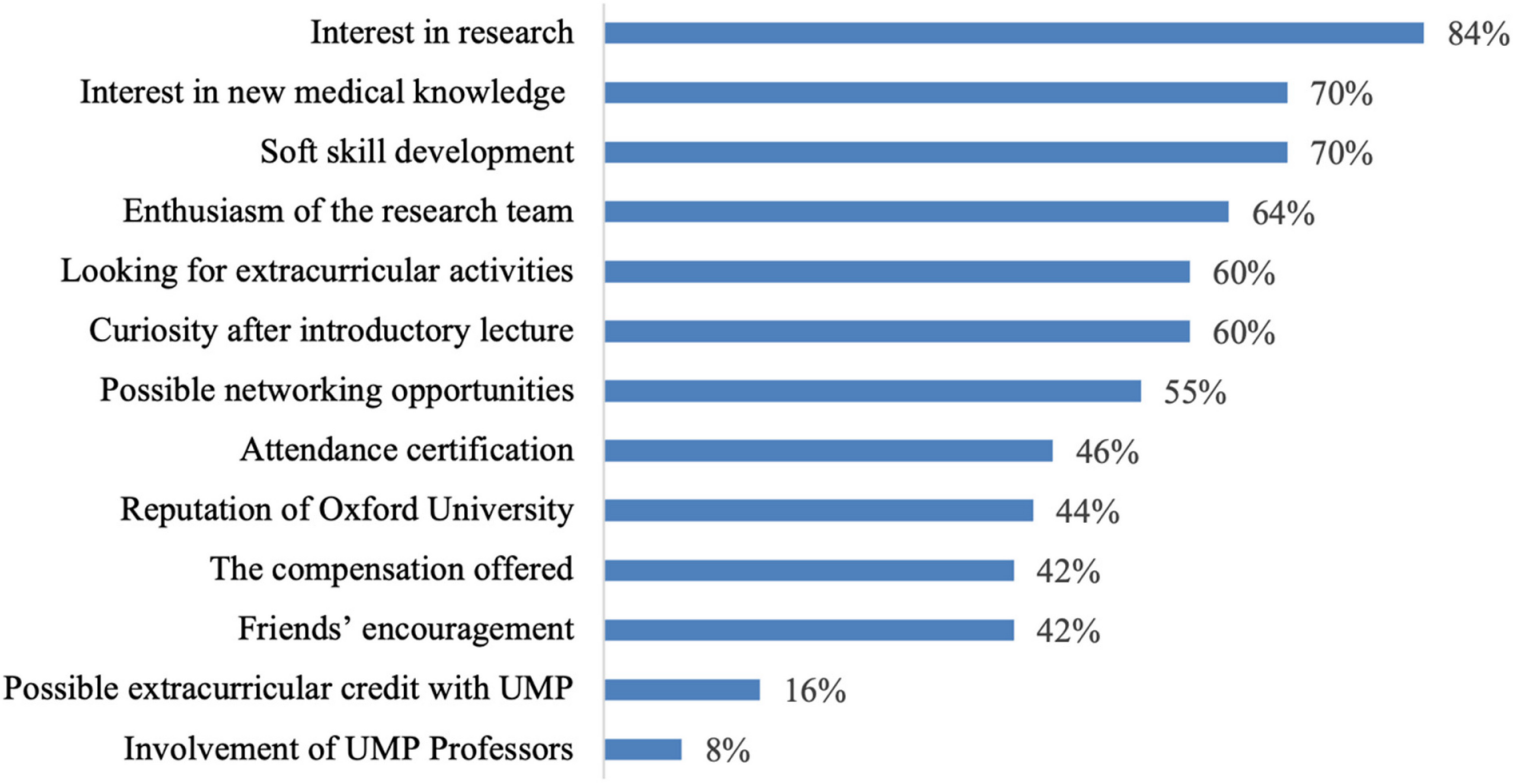
Motivations to join the SEED project, as reported by the 231 students who filled out a study completion questionnaire.

Students’ motivations to join the study are shown in Figure 3, ranked according to the frequency of selection on the completion questionnaire. Personal factors including an interest in scientific research (195/231, 84%), a desire to gain new medical knowledge (161/231, 70%) and a wish to improve their soft skills (161/231, 70%), were strong motivating factors for students to participate in the project. Other factors included the enthusiasm of the project team during the introductory sessions (148/231, 64%), which aroused the students’ curiosity while they were looking to engage in extracurricular activities (139/231, 60%), and the opportunity for networking (126/231, 55%). Factors such as the possibility of getting a project attendance certificate, the financial compensation offered, the reputation of Oxford University, and encouragement by their friends, were mentioned by smaller proportions of students (42–46%) as relevant to their decision-making.

These motivations were explored in more detail in the interviews. It was clear that the SEED project attracted considerable attention from students because of the novel format, incorporating scientific enquiry alongside enjoyable engagement activities. Students were also impressed by the well-prepared introduction and enthusiasm of the research team. These factors fostered a friendly and fun environment for students to become involved in the project and feel able to express their views freely.

*“Since the first event, I have seen that the program was prepared quite carefully, from the smallest detail, in every activity. The [research] team prepared pens, papers, tea breaks, cake at the events, and gifts as prizes for the competition activities. There were photos taken after every activity. That’s what impressed me the most.”* (G2_Pub5_IDI4)^1^

Other than their impressions of the introductory session, there were several important factors that influenced the students’ motivation to join, consistent with their expectations and how they perceived the benefits they might receive from participating in the project.

#### The pursuit of knowledge

Knowledge was the primary factor mentioned in the exit interviews that had motivated many of the students to join the project. They indicated that although scientific research had a significant impact on medical advances and was directly related to their field of study and future careers, they had limited knowledge about medical research and found the concepts unfamiliar and difficult. They anticipated that participation in the cohort would provide them with opportunities to gain more knowledge and experience about the research process.

*“Initially I wanted to participate in the project to try understanding what research is. I hoped, and I was also promised, to be able to learn more about research. This should help my work in the future, so I was quite hopeful about the knowledge that I might gain from this study.”* (G2_Pub5_IDI8)

After reviewing the preparatory material sent before each engagement event, any student could apply for a place depending on their personal interest in the discussion topic proposed and their availability. In general, the students appreciated the variety of topics and found the materials provided were of reasonable length and commensurate with their level of understanding. However, as indicated on the completion questionnaire, some students did encounter difficulties in understanding the topics, either because they did not have enough time to read the background materials in advance (29/221, 13%), or the topics were too difficult to understand (16/221, 7%). In addition, after joining several events some students, particularly those selected from Groups 2 or 3, told us that the topics started to overlap, or were outside their area of interest, and therefore they began to prioritize other activities instead of joining SEED events.

One issue raised by some students that affected their ability to gain maximal educational advantage from the project activities was lack of confidence. Although the students perceived that the research team used many measures to facilitate and encourage them to voice their opinions, in the completion questionnaire 66/221 (30%) respondents who attended an event admitted to at least one difficulty affecting their ability to participate actively in these events. The most frequent issue raised (by 42/66, 64% of these students) was that it was difficult to express their personal views in front of other people due to a lack of confidence. In the exit interviews possible contributing factors that were highlighted included differences in knowledge levels between freshmen and seniors, and between public health and general medicine students, and also the level of experience of students who joined many events compared to those who only attended occasionally. As a result, some students, especially those from G1, expressed difficulties in raising their opinions due to lack of confidence about their level of understanding, fears of being judged by their colleagues, or in some cases because they defined themselves as introverted and shy.

*“It [my involvement in the discussion] depended on the topic of that day. If I understood the topic and had prepared in advance, I would give my opinion. If I didn’t know anything about the topic, I chose to keep silent. /…/ I think those people [who rarely give opinions] are afraid of being wrong, afraid that other people would judge them as this or that.”* (G1_Med2_IDI15)

To address this issue students often preferred to join activities with their friends, who helped them feel more included and confident, and encouraged them to engage in discussions during events.

By the end of the program, the knowledge gained helped many students to have more confidence when discussing topics within the project’s scope, and some were even able to apply this knowledge more broadly to their university studies. To improve their understanding, several students expressed a wish to have more reference material provided, and to have more time to listen to the research team’s feedback and opinions at the end of each activity. The students also suggested deepening and diversifying the discussion topics in order to gain greater attention and improve retention rates, for example to cover research processes in more detail and explore other emerging infectious diseases. To enrich the group discussions, the students also proposed including other groups, such as those majoring in nursing or pharmacy, to gain multi-disciplinary views on ethics and medical research.

*“Later on, I thought that new knowledge is what attracts people to the program. For example, students who are in higher grades spend a lot of time studying. So, if we go to an event and gain knowledge that can serve our clinical work, it is more beneficial for us than coming to a discussion about basic [medical] knowledge.”* (G3_Med3_IDI13)

*“On the positive side, I think it is good because even when the topics were similar, each time our experience was different. Each time we were in a different group we discussed different issues, and we had different experiences. We looked at the problem from many aspects.”* (G2_Pub5_IDI4)

#### Improving soft skills

In addition to gaining knowledge, many students hoped to improve their soft skills through the various activities and interactions with researchers and colleagues. Participation in the project helped them to develop critical thinking skills and become more confident in communication, presentation, debating, and teamwork. Students who joined the poster and video competitions learned additional skills, including how to read and interpret scientific articles, infographic design, and how to make short but informative videos. Students in all three attendance Groups agreed that the promotion of soft skills development during the project was very helpful for their academic studies and their clinical practice, as well as for social activities.

*“When I had just entered my first year, I was very shy, I didn’t dare say anything. When working in a group, I was kind of “whatever,” and I just followed what the majority said. But after participating here [i.e., in the SEED project], when working in a group… I dare to express my thoughts. I also listen but… if I’m not satisfied with others’ ideas, I dare to refute them.”* (G3_Pub3_IDI6)

#### Networking and new experiences

By taking a different approach toward science that went beyond the typical academic curriculum, the students expected the SEED project to bring new experiences. They also aspired to become part of a community where they could develop their networks and learn from their colleagues. The diversity of school years and faculties involved in the project created space for them to expand their connections with other students as well as with the research team members. Some students commented that they had a chance to exchange experiences and knowledge with each other, and even swap tips on how to pass exams in school.

*“My expectation was to be able to meet more friends, outstanding ones… people say that when we interact with talented people, we become better too.”* (G1_Pub3_IDI11)

In a longitudinal project like SEED, participants’ experiences play a crucial role both in driving participation and prolonging retention. Positive experiences from previous events—such as active, enjoyable game-based activities followed by interesting discussions that facilitated a better understanding of science—motivated the students to participate in subsequent events. Furthermore, when the students volunteered to be team leaders, or took on a presenting or debating role, their experiences became more vivid and memorable. The more events they joined, the more they became aware of the different formats, were able to benefit from these new experiences, and were interested in trying more interactive approaches.

Conversely, unsatisfying experiences such as passively listening to a presentation similar to a lecture, boring discussions, ineffective interactive approaches, or feeling left out of their crowd of friends, discouraged them from joining subsequent activities. In such cases, the students considered prioritizing their time for their studies, part-time jobs, or alternative extra-curricular activities that might provide a better experience. These barriers were mostly raised by students who joined only 1 or 2 events throughout their involvement with the cohort.

*“Joining the sessions made me happy. There was nothing I wasn’t satisfied with. But right now, if I had to prioritize which program I wanted to attend, I probably wouldn’t choose SEED /…/ I saw posts on Facebook [about the events] saying the events were very enjoyable, the students were very connected, and I felt everyone’s excitement in that event. But when I came [to an event later], it didn’t seem to be like what I expected.”* (G1_Med2_IDI16)

#### Compensation

Compensation is usually not provided to students attending UMP extracurricular activities. The amount of compensation provided for attendance at SEED activities (equivalent to 10USD) was considered reasonable by 97% (211/218) of the students who responded to this question, although 3% (7/218) thought it was quite high compared to their basic life expenses or what they might earn for a similar time commitment to a part-time job. Students had different views about the impact of the compensation on their decision to participate in the activities. For most, neither the presence nor the amount of compensation had much influence on their decision to join the project. They prioritized the pursuit of knowledge and new experiences, as well as considering their potential contribution to the research effort.

*“God, I was shocked when I was first reimbursed [laughs]. I didn’t think that when I came here, I would gain knowledge, have my opinions respected, and even earn some money. So, I think it’s okay no matter how much it [the compensation] is. But for me, 200,000 [VND] is quite high.”* (G1_Med4_IDI18)

However, some students commented that compensation was one of the primary motivations for a small number of participants, including some of their friends. Based on their personal observations, students noted that participants who were motivated primarily by monetary benefit tended to be less interested and engaged in the research activities. As a result, the quality of the discussions tended to be reduced.

*“Since my friends also participated in the program, I know there were some who participated… for fun, for snacks, and for the money [smile], just the reimbursement, but not… not to gain knowledge. Just a few people, but there were some. These people often received emails [with event materials] but never read them, just left them there.”* (G3_Pub3_IDI6)

#### Students’ contribution to the project

Although we did not specifically ask the students’ views on their personal contribution to the project, thoughts about whether their participation had been meaningful were raised by some students in the exit interviews:

*“…I don’t know whether I have done anything meaningful for the research program.”* (G2_Pub5_IDI4)

*“When the team conducts research, you [the research team] must already know very well about those topics [research participant management, epidemiology], so I think my contribution isn’t very valuable. /…/ I find it [this research] takes a lot [of effort, time, money], but my contribution doesn’t have much value, and it makes me feel very sad. /…/ If I have another chance [to contribute] … I will be able to help the teachers [the research team]. But currently… my knowledge is still quite limited.”* (G3_Med3_IDI13)

One student expressed in the exit interview that she considered the relationship between her personal contribution (as she perceived it) and the level of compensation provided, as one of the barriers to her decision to join activities. Thinking of herself as someone with limited knowledge about the discussion topics, she felt that her contribution did not deserve the high level of compensation. Instead, she joined other UMP extra-curricular activities that did not provide compensation but that she felt were more suitable to her interests and to which she felt she had greater potential to contribute.

Instead of seeing their role as providing useful information to the research team, many students saw themselves as the primary beneficiaries of the research. In other words, similar to other extracurricular activities available to them, they considered that the SEED project was intended to provide students with knowledge, skills, and experiences relevant to clinical research rather than for the research team to collect data. However, one student commented that when considering medical ethics, collecting opinions from diverse groups of people (including their own) is quite important:

*“I feel that if it relates to medical ethics, we should listen to more opinions, from the youngest people to the oldest ones. Because each person has their own point of view. /…/ So, I think my position [as a participant] is quite important for scientific research. From what I’ve learned, I recognize the importance of medical ethics and people’s opinions toward ethical issues. When my opinions are collected, I feel that it is a very respectful thing.”* (G1_Med4_IDI18)

### Evaluation of organizational factors

In this section, students’ views on organizational factors related to the various project activities are summarized.

#### General

Among students who were motivated to join the SEED project, there were a number of barriers that prevented them from actually participating in events; 145/231 (63%) of the students who filled in the completion questionnaire mentioned at least one barrier to attendance. The most common reason cited by these individuals (127/145, 87%), was a busy schedule with studying and exams also emphasized in the interviews, especially for 3rd year students and above. Practical issues, such as difficulties with the location of in-person events, lack of transportation etc., were cited by some students as a barrier (20/145, 14%) but there were no significant issues with the topics offered, the format of events, or technical problems (≤ 6%).

Some students mentioned the participant selection process as a barrier. Due to restrictions on the number of students that could be accommodated at the different events we tried to prioritize places for those who had not previously attended any events. As a result, 41/145 (28%) of the students who mentioned a problem, generally those who had participated in several events, reported that they had received an email of refusal from the research team due to a high volume of applications for their chosen event. Some of these students expressed the view that being refused after registering to attend an event made them hesitant to sign up for future events.

#### Event organization

In the engagement events, the warm-up activities were appreciated by many of the students. These icebreaking activities were aimed at promoting interaction between the participants, thereby creating a relaxed environment in which they could later freely voice their opinions and ideas. Following suggestions made by some students on the post-event feedback forms during the early days of the project, the study team did in fact integrate more physical and competitive games into the initial phase of subsequent engagement events.

*“I’ve previously studied interdisciplinary education, so I see the important role of getting to know each other, sitting down together to connect /…/ If we didn’t play the warm-up games, I would be quiet from the beginning until the end, I would shut up like a clam, I wouldn’t say anything.”* (G1_Med4_IDI18)

The students also commented that having a range of different structures for the engagement events provided them with multiple options from which to choose, in accordance with their personal preferences. In addition, the changing format of the interactive activities kept them interested in the project over a long period of time. Many students liked the science debate and role-play formats because of their novelty and felt that the creative and competitive atmosphere helped them remember the content of the events afterward. These formats positioned them in circumstances where they were encouraged to explore conflicting views about ethical and social issues in medical research from different perspectives.

*“I found that the role-plays were more interesting. Participants would play a role [government employees, IRB members, healthcare workers, patients] in the scenario and act as if they were in a documentary. I found it easier to remember than the science debates.”* (G3_Pub2_IDI10)

Limited numbers of students were invited to participate in the FGDs and IDIs for formal data collection during the life of the SEED project, but these students also reported positive experiences with these activities. Some mentioned that by gathering in small groups of 4–6 students in the FGDs, or one-on-one with a researcher in the IDIs, they had additional time and space to explore their personal views on the specific topics discussed more generally during the engagement events. Through this process, they felt that they gained more insight, both from others, but also from carefully considering their own viewpoint.

The majority of the events were conducted offline, except between 2020 and 2021 during the COVID-19 pandemic, when many activities were converted to an online platform. Most students preferred participating in-person, citing the vibrant atmosphere and possibility of physical interactions (games, etc.), but some also mentioned the enjoyable tea-breaks. These factors made the students feel more open to participating in the discussions, facilitated networking, and meant that the information presented to them was more memorable. However, the large number of participants attending each offline event, (typically 2–4 groups of 15–20 students/group were present), limited the opportunities for individual students to raise their opinions. Students proposed either dividing the attendees at an event into a greater number of smaller groups (5–10 students/group) or increasing the time for discussion and student-led presentations. At the same time, they felt it was necessary to balance the time allocated for games, discussions and presentations, with the need for breaks during the events (that might last 2–3 h overall), as well as the need to finish on time to minimize fatigue and avoid compromising the participants’ attendance at other commitments.

Inevitably, during online events there were some limitations to effective engagement, particularly with respect to coordinating physical activities and the discussion sessions. Although online events were more comfortable for some students and were often viewed positively as more convenient in terms of time and location, other students were reluctant to participate in online events. Overall, considering the 40 sign-up engagement events included in this analysis (i.e., excluding open-access events and specialized invitation-only events), an average of 51 students joined each of the 30 offline events, while an average of 33 students joined each of the 10 online events. During many of these events, small numbers of students were elected or volunteered for important tasks such as leading a debate or contributing to a role play scenario. While this was generally a positive experience for the selected individuals, some other students felt discouraged from contributing actively to their team responsibilities. As a result, in addition to reduced satisfaction among these “inactive” students, the overall experience of some of the more enthusiastic students was also adversely affected.

*“The thing is, the issue was the same [as in previous events], people who were not the designated debaters did not participate in the group discussion. I found it quite sad. I only scored the event 7/10 [of satisfaction]. I don’t like the fact that those people had no responsibility to the group and were less involved [in the discussion].”* (G3_Med5_IDI1)

#### Communication

In addition to email, we utilized Facebook and Zalo, the most popular social media platforms in Vietnam, to reach the maximum number of potential participants for the various cohort activities. The majority of students found it easy to see announcements for new events and received scientific information regularly in the form of papers, videos, or infographics. Students also suggested posting monthly topic calendars and summaries of previous engagement events to make it easier for them to follow the discussed topics and arrange their schedules. To design activities and communication strategies more suited to young people, we also involved small teams of 6–8 student ambassadors who helped us develop content and operate the online engagement using youth-friendly approaches. The ambassadors were proactive cohort participants, selected following a competitive application process, with each group supporting the research team for approximately 6 months.

However, communication via social media was still evaluated as a weakness of the project. Students mentioned that a low interaction rate on social media was also an issue for other UMP activities and commented that more creative approaches could be used to gain attention. Due to the volume of information presented generally on Facebook, some passive cohort participants paid little attention to the research and scientific information they received. Several students recommended that outputs from the more creative project activities, such as the scientific posters and videos generated by students, could be used as promotional materials to capture the attention of cohort participants more effectively. In addition, this approach might help disseminate knowledge of the SEED project to other students across the university, who might not otherwise be aware of the project.

### General views on the SEED project and scientific research

**FIGURE 4:**
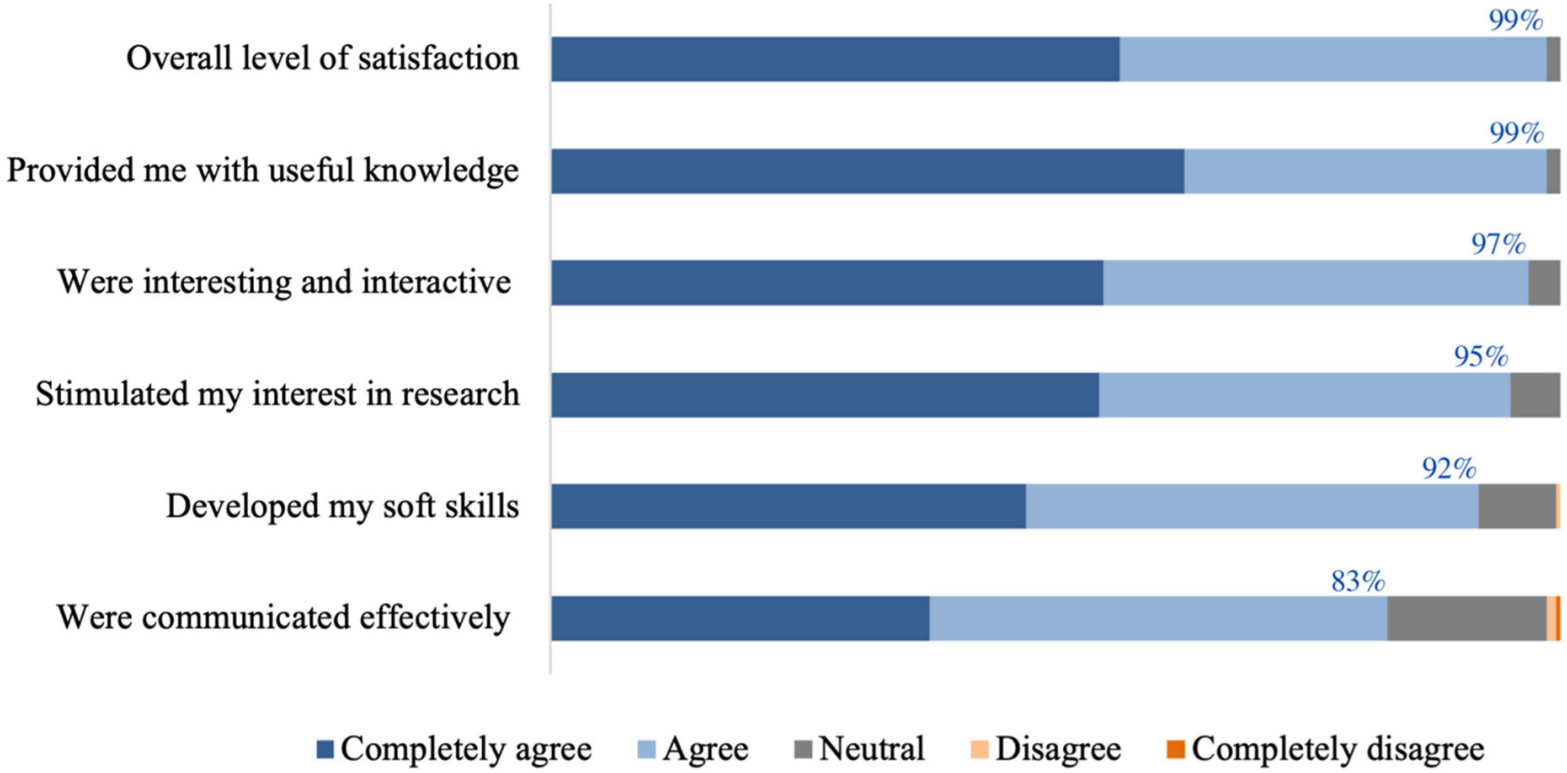
Students’ general views about the SEED project activities (*n* = 221).

**FIGURE 5:**
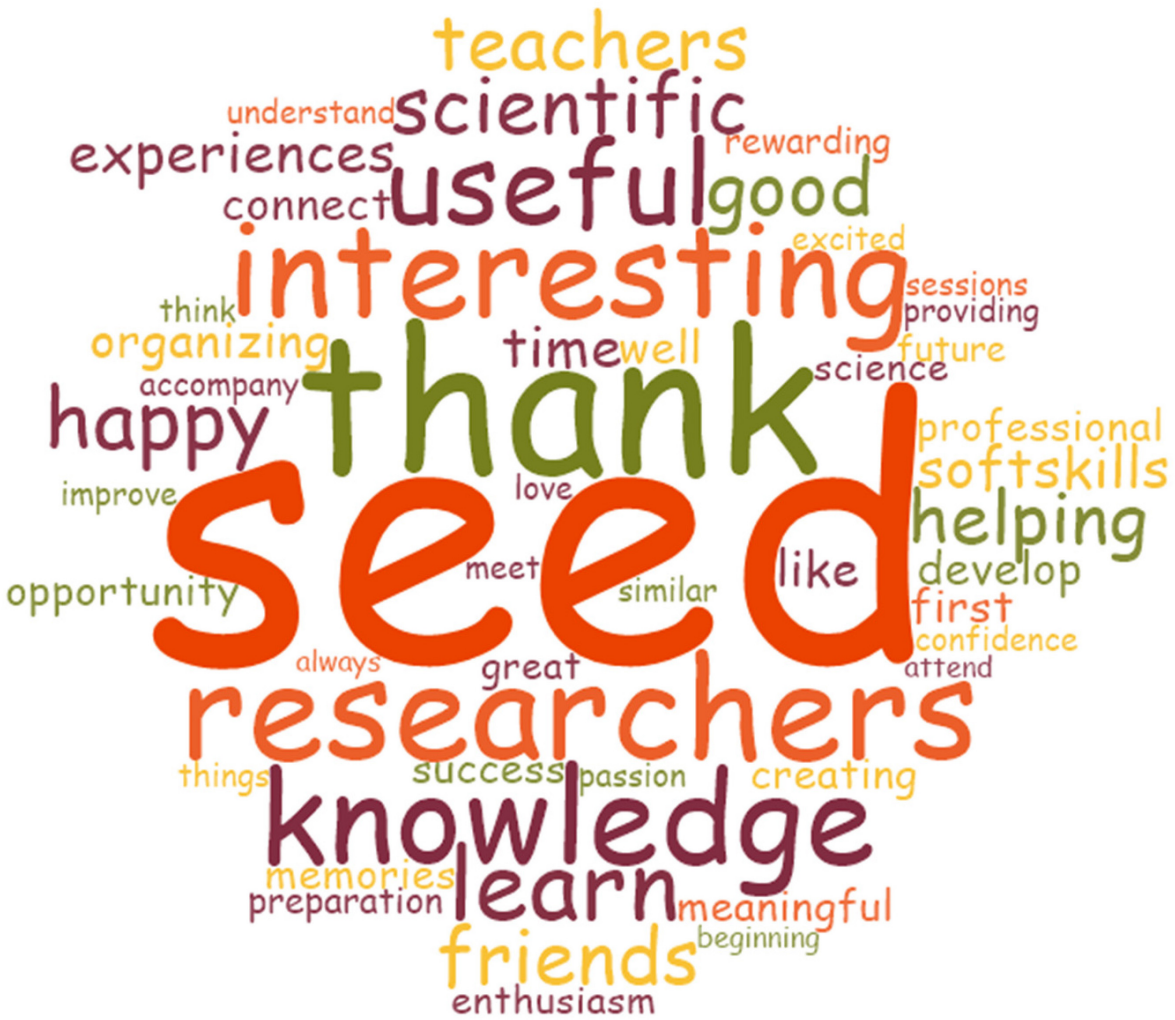
Word map summarizing comments about the SEED Project, collated from free text responses by 231 students on the study completion questionnaire.

For most students, joining the SEED project was the first time they had played an active role in discussing medical research. Figure 4 shows the students’ responses to questions regarding their overall assessment of the project and Figure 5 displays a word map summarizing their comments on the completion questionnaires.

Almost all students who responded to this question (217/220, 99%) agreed (completely agree/agree) that they were satisfied with their participation in the project. It was a meaningful program, both for their studies and for their future careers as healthcare workers or researchers. Scientific research had been an unfamiliar and “arid” topic, but after participating in the SEED project, it became more interesting as they were encouraged to carefully consider research in the context of various complex issues related to the practice of medicine, ethics, eco-social factors, the community perspective, and public health policy. The more they learned, the more they understood the complexity and importance of strict regulation for the conduct of clinical research. Through interactions with the research team, the students became increasingly curious and began to understand the necessity for research in both high-income and low/middle income contexts, especially on infectious diseases. For example, dengue is a common, potentially fatal viral infection, the pathogenesis of which is poorly understood and for which no specific therapy is yet available; at least one individual was inspired to consider undertaking research on this topic in the future.

*“I see that the teachers [researchers in the project] really want to spread the fire [passion] to us. Giving us positive energy to study hard, to contribute to society, and to do research. /…/ I see research in Vietnam… is not really well developed. Our economic conditions don’t allow people to devote themselves wholeheartedly to research. But I see that the teachers [researchers in the project] are very passionate about this work. Being in contact with researchers in this way will motivate me more to do research.”* (G3_Med3_IDI13)

*“After participating in this event, my intention to choose dengue as a topic for my undergraduate thesis has become stronger and stronger.”* (G1_Pub3_IDI11)

Furthermore, in addition to learning about clinical trials for new medicines and about the contribution of laboratory science to improving knowledge about diseases, students also gained a better understanding of social science research. They came to recognize the importance of exploring the perspectives of all relevant stakeholder groups on health issues that affect a community, as well as listening to the views of experts. Students also commented that the deliberative engagement approach used by the project has great potential to effectively involve students and the wider public in scientific discussions, making it a method that should be more widely adopted.

Lastly, students appreciated the careful preparation for each event and the enthusiasm of the research team, thereby creating a welcoming, interactive, and open space where they felt able to express their opinions freely without judgment.

*“I want to thank the project for creating opportunities for medical students to have interesting experiences and to gain many skills and knowledge during school hours, to support our studies as well as our work in the future.”* (G3_Med2_IDI5)

*“SEED project is an opportunity for me to step out of my comfort zone, a scientific and social environment full of potential for us to develop.”* (Completion questionnaire)

*“It was the first time I participated in an academic project. When participating in the project, I found the research team enthusiastic and thorough with this research, including physicians and non-physicians. As for me, I could state my thoughts from what I know, without being judged right or wrong. The Science Debate was logical and most of the ideas were personal opinions. The important thing was that I felt our points of view were recognized. After the explanation [about the discussion topics] from the research team, I gained more accurate information and understood everything more thoroughly. In addition, the project also had a lot of gifts, and even prepared food for us. There was so much care! Although I did not participate much in the project, I really thank the project very much!”* (Completion questionnaire).

## Discussion

Although evaluation is considered to be a critical process for assessing the effectiveness and impact of engagement programs, in many youth engagement activities related to health research in LMICs formal evaluations remain limited (9). Our study, conducted during the last 6 months of the 3-year SEED project using a variety of different tools, has provided valuable insights into university students’ motivation to participate in a health-related social science research project, while also exploring their experiences throughout the process. However, since < 50% of the SEED project participants responded to the study survey and students who participated readily in the exit interviews tended to be from among the more active cohort members, we acknowledge the risk of selection and positivity bias when interpreting our findings.

Following the structure of Figure 2 we present the discussion of our findings in terms of positive and negative factors associated with student participation, as well as a section focused on these potential biases.

### Motivations to join, perceived benefits and positive experiences

Personal benefits, such as gaining new knowledge, enhancing soft skills, and exposure to networking opportunities, were major motivating factors, but financial compensation was not considered very important by most students. Our study aligns with previous research indicating that healthcare students are highly motivated and eager to participate in activities that enhance their medical knowledge, professional competencies, and soft skills, particularly those that allow them to contribute in some way to the wider community (31–33). While the students in this study often engaged in scientific activities through UMP, these interactions tended to be passive and brief. The SEED project provided a novel platform for students interested in medical research, offering opportunities to expand and apply their academic knowledge through interactive engagement with their peers and recognized experts. Given the growing demand for multidisciplinary medical education, the skills acquired through the project, such as communication, teamwork, critical thinking, and the ability to present and defend ideas supported by evidence, held significant value for students’ future careers, particularly in contexts where such skills are not typically emphasized in academic programs (10, 31). Moreover, the project encouraged students to consider medical research within a broader and more practical framework, integrating ethical and socio-cultural dimensions of health issues and community programs. These insights may better equip students to contribute to interdisciplinary health initiatives that require active community participation (34).

Besides the motivation to participate in the SEED project for self-development, a few students in the study mentioned their desire to contribute personally to the development of science and provide benefits to their communities from their own knowledge and experience. Students who felt their contributions were valued, particularly when their perspectives on the research topics discussed were acknowledged, reported positive experiences; in contrast, others (including quite active cohort members in G2 and G3), reported feeling discouraged from ongoing participation when they perceived the value of their contributions as unclear. Altruistic motivation has been mentioned in other studies with young people (35, 36). In clinical trials the outcomes are often tangible, as volunteers’ participation can contribute to the development of new vaccines, therapeutics or other interventions (37, 38). Conversely, in social science research, the outcome is typically an enhanced understanding of community perceptions. Considerable challenges still exist when attempting to translate such insights into tangible impacts on healthcare systems, and thus the external visibility of the participants’ contribution may be limited. Based on this work and other studies, when participants perceive the impact of their contributions to be small (regardless of more immediate benefits such as personal remuneration), their motivation to continue may be diminished, potentially leading to lower retention rates (35). Proactively involving study participants in disseminating research outputs to relevant communities could help to foster an understanding that their contributions have value, thus enhancing their enthusiasm for and engagement in such activities (10). This phenomenon may also explain why students are drawn to extracurricular activities like volunteering in remote areas to teach children or provide free healthcare, as these activities offer a sense of accomplishment through benefiting others in the community (35, 39). Several strategies should be considered to enhance the visibility, tangibility, and impact of such contributions; these include regularly reminding participants of the research objectives and goals, acknowledging and attributing volunteer contributions (36), designing activities that are led by participants, and creating communication products to disseminate research results to the broader community (10, 16).

Organizational factors also emerged as important contributors to positive student experiences during the SEED project. Incorporating diverse engagement activities, such as science cafés, debates, role-playing, and poster and video competitions, provided students with multiple opportunities to participate in formats aligned with their preferences. This diversity appeared to be a successful approach for enhancing student participation and maintaining their interest throughout the program. In particular, offline events, which allowed for more physical and interactive forms of engagement, attracted higher average attendance and were viewed more positively by most students than online events.

### Barriers to participation and negative experiences

In addition to considering students’ motivations and interests when designing research and engagement initiatives, the study highlights the importance of regularly assessing barriers that may hinder participation. Common barriers reported here and in previous research studies include conflicts with the students’ academic schedules, limited event capacity affecting the number of participants with high interest (31, 32), and repetitive or unengaging topics. Furthermore, although social media is central to youth engagement, our project did not leverage it very effectively. Project announcements on platforms like Facebook were easily ignored, particularly among students with less interest, who either did not participate in any activities, or had had negative experiences at previous events.

In order to increase the interactions on social media we made use of student ambassadors, applying a recognized strategy that involves young people in co-designing media content (40–42). Youth often bring valuable insights into peer engagement, including regarding preferred content, interaction patterns, attention span, and creative design approaches (41). Youth-led campaigns have been shown to achieve high impression and engagement compared to professional campaigns and to improve knowledge, perceived risk, and motivation for action among peers (40, 43). Additionally, social media platforms’ metrics can be utilized to evaluate the level of interactions (numbers of clicks, comments, likes or reactions, shares) and understand which topics, types of activities, and media products generate the most engagement (42). However, although the student ambassadors themselves reported high levels of satisfaction, we did not formally evaluate the effectiveness of their initiatives or the use of social media analytics. Future studies should incorporate these approaches to strengthen communication strategies, adapt engagement methods, and support ongoing evaluation.

Our evaluation identified several other factors associated with negative student experiences. Students who participated in many activities sometimes found the discussion topics repetitive, while many students perceived online events as passive and less interactive. In contrast, some less confident students reported feeling uncomfortable speaking up or isolated during offline events and considered online settings easier for contributing to discussions. The shift from offline to online activities in our project was driven by the COVID-19 pandemic and associated social distancing restrictions. COVID-19 substantially changed the way education, work, and extracurricular activities were conducted during and after the pandemic. Although online events may improve accessibility, they also present challenges commonly reported in youth engagement settings, including limited face-to-face interaction when participants keep cameras off, unstable or limited internet access, and difficulty assessing students’ engagement during sessions (44).

As a result, online engagement activities require creative strategies to maintain participation. Suggested approaches include providing clear instructions for using online platforms, addressing technical issues early, encouraging interaction through chat functions or emojis, and promoting camera and microphone use by emphasizing the value of students’ voices and participation (44). A range of digital tools may also support engagement, such as photovoice or video for conveying information (45, 46), Kahoot, Quizizz, and Mentimeter for quiz-based competitions, or Padlet and Jamboard for group discussions. Depending on the target audience and context, research teams should collaborate with young people to design activities that ensure alignment of the research themes, discussion topics, and appropriate engagement methods. For longitudinal studies a process of ongoing review, evaluation and adaptation is important to improve the students**’** overall experience and encourage diverse engagement strategies that foster an open environment for participation.

### Limitations

Despite trying to optimize the tools and methods used for data collection during our evaluation process, certain shortcomings and areas for improvement remain. First, our assessment was limited to the student participants alone; due to time and resource limitations we were unable to collect similar data from members of the research team or from our collaborators at UMP. Such assessments could have provided important information from the organizers’ perspective about working with students and any challenges encountered during the research process, as well as potentially providing insights into possible improvements for future research efforts.

Second, response rates were low from students who were less engaged in the project activities for both study completion questionnaires and exit interviews. Despite repeated efforts we were only able to obtain responses to the completion questionnaire from a small sample (*n* = 10) of students who had not participated in any project activities (G0). Consequently, the findings lack substantial insight into how to enhance engagement with this group or identify motivations that might encourage their participation in research. Additionally, the low acceptance rate (43%) for interviews from G1 students (i.e., those who participated in only one to two project activities), may indicate a similar reluctance among this group to share their experiences, potentially due to negative or critical perceptions of the project. Thus, although we received predominantly positive feedback from students, we acknowledge that various factors may have influenced this finding. Students who declined the interview invitation may have had more negative experiences, while those who participated and perceived themselves to be beneficiaries might accept or ignore certain limitations of the project activities. Additionally, the interviews were conducted within the Vietnamese cultural context, where criticism is often avoided (47). This tendency is amplified by the hierarchical “students vs. teachers” relationship, which demands great respect in Asian cultures. Finally, although the familiarity between interviewees and interviewers, developed over a prolonged period of interaction, may have encouraged openness in sharing experiences, this familiarity could also have increased reluctance to provide negative feedback to avoid offending the research team. To mitigate this potential source of bias the surveys and semi-structured interview guides were designed with neutral wording and a balance between positive and negative questions. In addition, the interviews were conducted by experienced staff trained in both social science and public engagement, aiming to foster a friendly but honest atmosphere that allowed the students to make critical comments should they wish.

## Conclusion

There is growing emphasis on formal evaluation of healthcare-delivery and educational programs in order to support and promote effective utilization of resources. Similarly, incorporation of multidisciplinary impact assessments is now a standard requirement for many research grant funding bodies. As yet however, few publications have addressed evaluation of social science research programs, especially in LMIC settings. In this manuscript we present a mixed-methods participant-centered evaluation of the experiences of Vietnamese healthcare students enrolled in a longitudinal cohort study focused on the conduct and ethics of clinical research. The evaluation emphasizes the importance of designing and adapting engagement approaches based on feedback at multiple time points, integrating different forms of activities, identifying and addressing difficulties and barriers preventing participation, and highlighting the social and scientific contribution of students’ voices in enhancing their experiences and retention. Future studies should include more rigorous evaluation of the impact of collaborating with young people using the co-design approach, and the effectiveness of social media communication in improving participants’ experiences and engagement.

## Data Availability

The quantitative data supporting the conclusions of this article will be made available by reasonable request to the OUCRU Data Access Committee at dac@oucru.org.

## Footnotes

1 The quotes in this paper are accompanied by codes specific to the interviewee. The codes summarise the interviewee’s information, including Group (G1, G2, or G3), Faculty (Medicine or Public Health), academic year, and a code number representing their in-depth interview. For example, “G1_Med5_IDI9” indicates the interviewee is in Group 1, is a 5th-year student at the Faculty of Medicine, and their in-depth interview was the 9th conducted.
