# Supplementary material for: Evaluating the experiences of Vietnamese university students enrolled in a longitudinal research cohort: a journey mapping approach
Source: Front Med (Lausanne). 2026 Jun 8;13:1745499. doi: 10.3389/fmed.2026.1745499 (PMC13283861; doi:10.3389/fmed.2026.1745499)
Supplement: Supplementary file 1 [file Table_1.docx]

# Appendix 1. Exit interview guidance

**MAPPING STUDENTS’ JOURNEYS**

Each interview is expected to last around 45 to 60 minutes. The sessions include 2 main parts:

Experience map creation

Semi-structured interview

**Experience map creation**

- At the beginning of the interview, the participant is asked to create their own experience map. Using the experience map technique allows the student to reflect on their experiences and show the interviewer their journey, centered from their perspective. This map is then used throughout the interview to guide the conversation.
- To create these maps, the student is given a blank piece of paper with a horizontal line separating sections indicating positive and negative experiences, and a vertical line representing the timeline during their participation in the SEED project. They are also given a set of sticky notes naming the different types of activity. They are asked to select notes representing the different activities that they joined, and to place these notes on their map to create a timeline of their participation.
- Students can use other stickers to add more details about the activities such as topics, formats, and their positive or negative experiences.

**Semi-structured Interviews**

- After the student creates their individual experience map, we proceed to a semi-structured interview. There are standard questions to start and end the interview.
- The interviewer then uses prompts to initiate a conversation to learn more about the individual’s experiences.
- The end of the interview includes big picture questions on the student’s overall thoughts on the project activities.

Follow-up questions after experience map creation (pick any questions from the list)

- Can you walk me through the map you created?
- From the experiences you listed, can you choose which ones you would like to talk about?
- I see that you listed [x experiences] above and below the timeline? Can you explain why?
- Which activities did you feel were the most rewarding/challenging? Can you tell me a little bit more about those experiences?
- Did you find any benefits throughout the project? And any challenges?
- What do you think about your own role/contribution to the project?
- What do you think about the level of reimbursement for each event?
- From your personal experience, what do you think we could do to improve the project?
- Is there anything else about your journey you would like to share today?

Interviewers may also ask additional questions to investigate further about particular activities that students participated in while they are describing their map.

The end of the interview includes big picture questions on the student’s overall thoughts on the SEED Project:

|  | **Pre-experience** | **Post-experience** |
| --- | --- | --- |
| **Motivation** | What motivated you to join these activities? | What were you motivated to do after you completed these activities? |
| **Learning** | What were you hoping to learn during the project? Was there anything you expected to learn during the project? | Could you describe one or two lessons from this experience that have influenced you after you completed project? |
| **Skills** | What skills did you expect to gain from this project? | What skills did you gain? How have these skills allowed you to pursue other things? |
| **Expectation** | What was your overall expectation before joining the project? | Do you think your expectations were met after joining these activities? |

**Example of an experience map created by a student**

**
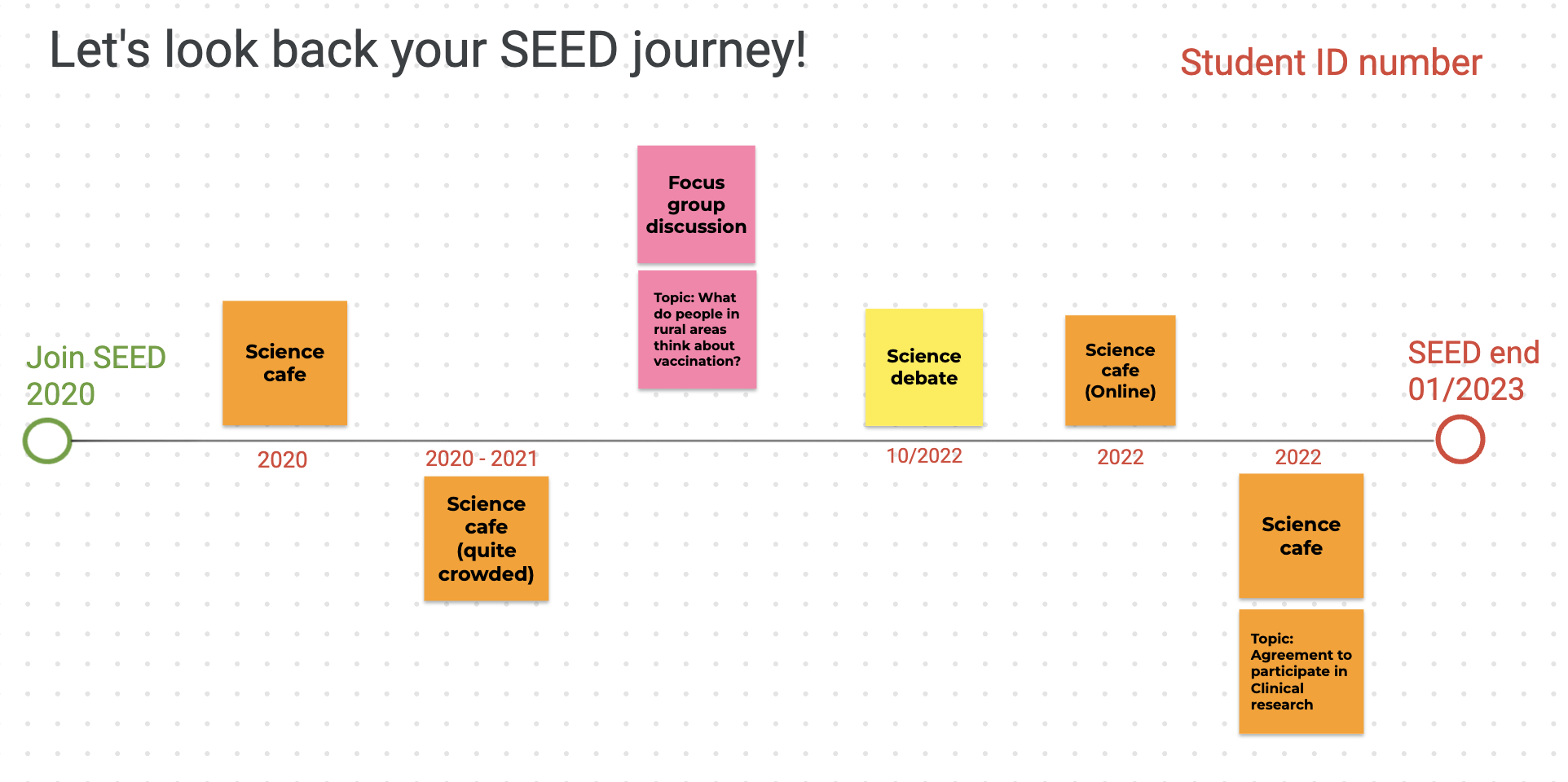
**

(Difficult to understand)

(Excited)

Negative

Positive

#

# Appendix 2. Completion questionnaire for research evaluation

**SEED - COMPLETION QUESTIONNAIRE**

Thank you for participating in the Seed project. We would very much like to hear your thoughts about the SEED project, now that the project is coming to an end. Please fill out this form, it should only take a few minutes. All the information you provide will be stored securely following our standard procedures.

1. Study code: *(required question)* 55DX-113-[__|__|__]
2. Date of completing this form [dd/mm/yyyy]
3. What made you decide to enroll in the SEED Project originally? *(You can choose more than one answer)*

My interest in scientific research

My interest in gaining new medical knowledge

To develop my soft skills

I was looking for enjoyable extracurricular activities

I was looking for possible networking opportunities

I was curious about the project after the introductory lecture

The enthusiasm of the research team

Friends invited/encouraged me to join

Our professors were the project’s PIs

The reputation of Oxford University

I thought I might get extracurricular credit with my university

The possibility of getting an attendance certificate

The compensation offered for attending SEED events

Other reasons (Please share): …

1. Please let us know your thoughts about the study activities of the Seed project.

(Please note: “Totally agree” on the left and “Totally disagree” on the right)

| **SEED activities …** | **Totally agree**  **😃** | **Agree**  **🙂** | **Neutral**  **😐** | **Disagree**  **🙁** | **Totally disagree**  **😠** |
| --- | --- | --- | --- | --- | --- |
| Provided me with useful knowledge |  |  |  |  |  |
| Stimulated my interest in scientific research |  |  |  |  |  |
| Developed my soft skills |  |  |  |  |  |
| Were interesting and interactive |  |  |  |  |  |
| Were communicated effectively |  |  |  |  |  |

1. What do you think about the amount of financial compensation provided in relation to your effort when you participated in a SEED activity?

☐ Too much ☐ Right amount ☐ Too little

1. What is your level of satisfaction with the SEED project overall (i.e., considering all the activities that you have participated in)?

☐ Very satisfied

☐ Satisfied

☐ Neutral

☐ Dissatisfied

☐ Very dissatisfied

1. Which study events did you join (*tick all event types that you remember attending*)?

☐ Science café

☐ Science debate

☐ Role play

☐ Video Competition – PAR

☐ Poster Competition

☐ Focus group discussion

☐ In-depth interview

1. Among the events that you remember attending, which format impressed you the most? *(One answer only)*

☐ Science café

☐ Science debate

☐ Role play

☐ Video Competition – PAR

☐ Poster Competition

☐ Focus group discussion

☐ In-depth interview

1. Did anything stop you from attending any SEED activities after you had enrolled? ☐ Yes ☐ No

If Yes, what were the factors? *(You can choose more than one answer)*

I was too busy / did not have time to attend

The event formats were not attractive to me

The topics were not of interest to me

I did not receive any information/announcements about the events

Practical problems – e.g., difficulties with event locations, lack of transportation, etc.

☐ Technology issues – e.g., difficulties with my computer, wi-fi access, online tools

☐ I registered to attend the events, but my requests were not successful

Other reasons (Please share): …

1. Were there factors that affected your participation during events that you did attend? ☐ Yes ☐ No

If Yes, what were the factors? *(You can choose more than one answer)*

☐ The topics discussed were too difficult to understand

☐ I did not have time to read the relevant references/materials in advance

☐ The time for discussion during the event itself was too short

☐ Practical problems during an event – e.g., too many participants, too noisy

☐ Technology issues during an event – e.g., difficulties with my computer, wi-fi access

☐ I found it hard to express my views in front of other people (lack of confidence)

☐ Other issues (Please share): …………………….

1. Could you please share more about your feelings and thoughts about the project?

# *Appendix 3. Post-event feedback form*

**VOLUNTARY FEEDBACK FORM**

We are very interested to hear your opinions about any activities that you attend related to the SEED project. If you are happy to give feedback please complete this form, which is completely anonymous. We will store the data securely in line with our normal procedures, but it is not possible for any information you provide to be traced back to you.

Date of Activity: __/__/___

| **Items** | **Completely Agree** | **Agree** | **Do not know** | **Disagree** | **Completely Disagree** |
| --- | --- | --- | --- | --- | --- |
| The content was useful for my personal knowledge | 🞎 | 🞎 | 🞎 | 🞎 | 🞎 |
| The content stimulated my interest towards clinical research | 🞎 | 🞎 | 🞎 | 🞎 | 🞎 |
| The session was fun and interactive | 🞎 | 🞎 | 🞎 | 🞎 | 🞎 |
| The content met my expectations | 🞎 | 🞎 | 🞎 | 🞎 | 🞎 |
| I would like to join other sessions | 🞎 | 🞎 | 🞎 | 🞎 | 🞎 |

How do you feel generally about the activity? Please give any additional thoughts or comments in the box

What could be done to improve the activity?

If you are happy to be contacted to discuss your feedback in more detail, please write your name, phone number or email address in the box below. A member of the study staff may contact you later, but you are not under any obligation to answer their questions unless you wish to.

# Appendix 4. Characteristics of the participants who attended an exit interview

*Table S1. Number of invitations and acceptance rate of exit interviews.*

| **Group** | **Number of Students** | **Number of Invitations** | **Number of Interviews** | **Acceptance rate** |
| --- | --- | --- | --- | --- |
| (G1) 1 – 2 events | 152 (34%) | 14 | 6 | 43% |
| (G2) 3 – 5 events | 159 (35%) | 6 | 6 | 100% |
| (G3) 6 – 16 events | 141 (31%) | 6 | 6 | 100% |
| **Total** | **452** | **26** | **18** |  |

*Table S2. Demographic characteristics of students who attended an exit interview*

|  | | **Exit Interviews**  **(n=18)** | | **All cohort***  **(N=539)** | |
| --- | --- | --- | --- | --- | --- |
|  | | **n** | **%** | **n** | **%** |
| **Gender** | Male | 8 | 45 | 271 | 50 |
|  | Female | 10 | 55 | 268 | 50 |
| **Faculty** | General Medicine | 12 | 67 | 365 | 68 |
|  | Public Health | 6 | 33 | 174 | 32 |
| **Year** | 2^nd^ | 4 | 22 | 100 | 19 |
|  | 3^rd^ | 5 | 28 | 189 | 35 |
|  | 4^th^ | 1 | 6 | 48 | 9 |
|  | 5^th^ | 8 | 44 | 143 | 27 |
|  | 6^th^ | 0 | 0 | 59 | 11 |
| **Interview format** | Offline | 6 | 33 | _ | _ |
|  | Online | 12 | 67 | _ | _ |

** Includes 10 students who withdrew from the cohort, and 77 students who did not attend any events/activities.*
